# The Economic Cost of Burnout in Veterinary Medicine

**DOI:** 10.3389/fvets.2022.814104

**Published:** 2022-02-25

**Authors:** Clinton L. Neill, Charlotte R. Hansen, Matthew Salois

**Affiliations:** ^1^Department of Population Medicine and Diagnostic Sciences, Center for Veterinary Business and Entrepreneurship, Cornell University, Ithaca, NY, United States; ^2^Veterinary Economics Division, American Veterinary Medical Association, Schaumburg, IL, United States

**Keywords:** burnout, economic cost, veterinarian, veterinary technician, sensitivity analysis, mental health

## Abstract

The purpose of this study is to estimate the economic cost of burnout in the veterinary profession and highlight the financial reasons why the industry should address the burnout crisis from an organizational perspective. Using data from 5,786 associate veterinarians in private practice, information was obtained using employment information related to compensation, work hours, hour preferences, and job turnover. Burnout was measured using the Professional Quality of Life Scale and used to calculate conditional probabilities on turnover and reduced working hours due to burnout. Lost revenue from each outcome (turnover and reduced working hours) was then used to calculate the economic costs to the veterinary services industry. The attributable cost of burnout of veterinarians to the US industry is between $1 and 2 billion annually in lost revenue, though there is a large amount of uncertainty. The cost is dependent on whether veterinary technicians are included in the analysis. The highest economic cost per veterinarian is among food animal practitioners, while the lowest is among equine. This study demonstrates that there are significant economic costs due to burnout among veterinarians and veterinary technicians. We suggest pursuing organizational interventions as these have shown the most impact in decreasing burnout and increasing satisfaction among human health physicians.

## Introduction

Burnout is a prominent problem in many healthcare professions (1–4) and not solely unique to veterinary medicine. The World Health Organization defines burnout as an “occupational phenomenon” and is characterized as a syndrome resulting from chronic workplace stress (5). In a recent survey, 86.7% of US veterinarians had ProQOL burnout scores in the moderate or high range (6). This trend of burnout is increasing over time (6, 7), as well as overall stress levels for veterinarians (8). Burnout not only affects the psychological and emotional health of individuals, but also the economic health of practices and the entire industry (9). In addition, the COVID-19 pandemic added additional burdens to veterinary staff that likely exacerbated the causes of burnout such as changes in operations (shifting to curbside care service, social distancing practices, increased sanitation measures, personal protective equipment adoption, and adopting new technologies such as contactless payments, virtual check-ins and telemedicine), and having to adjust to drug and staff shortages, and sick employees (10–13). This study is the first to quantify the economic impact of burnout in veterinary medicine.

Burnout in physicians is largely driven by excessive workloads, imbalance between job demands and skills, a lack of autonomy, and prolonged work stress (2, 14). While everyone is susceptible to burnout at some level, it is even more so with those in the frontline of the veterinary profession (1, 15). Among psychologists, working longer hours contributed to higher emotional stress; respondents in solo or group independent practices reported a greater sense of personal accomplishment, more sources of satisfaction, fewer sources of stress, and more control at work than respondents in agency settings. In addition, women in independent practice reported less emotional exhaustion than women in agency settings (16, 17). In the veterinary industry, studies have shown that burnout is higher among recent veterinary graduates (6, 18), veterinarians with high veterinary educational debt (6), those who spent most of their time with cats and dogs (6) and women (6, 19, 20). Burnout is also more prevalent among associates in private practice compared to practice owners, relief veterinarians, and veterinarians in public practice (roles in academia, government, non-for-profit, industry, etc.) (7).

From an economic perspective, higher rates of burnout affect both sides of the market—lower consumer and producer (veterinarian) welfare. While there is a lack of research in the veterinary profession, it would not be out of place to state that burnout also affects a veterinarian's ability to attend to patients effectively and efficiently, which lowers an individual's earning potential, as well as the clinic's profitability (9, 21, 22). Without addressing the issue from an organizational perspective, the responsibility of managing burnout is left to the individual veterinarian. While individual actions, such as maintaining a balance between personal and professional life, have shown to decrease emotional stress (23), industry-wide actions might more effectively address the increasing rates of burnout. Specifically, organizational interventions have shown to be much more effective at decreasing rates of burnout among healthcare professionals (15) and should be the focus of future work on burnout in veterinary medicine.

The economic impacts of burnout are predominately measured *via* turnover and reduced working hours (9). Turnover is an important characteristic to measure because a clinic will experience lost revenue due to a reduced labor force. In addition, there are search and training costs with finding a replacement (24). Search costs are the expenditures associated with marketing/advertising, interviewing, recruiting, and other associated activities with finding a veterinarian to replace one that exited a practice. Intention to reduce or an actual reduction in working hours also leads to lost revenue for a clinic (9). Moreover, it was found that physicians who are experiencing burn out and working more hours than desired often have more self-reported medical errors and poorer patient outcomes (21). So, even an intention to reduce working hours due to burnout among veterinarians means that clinics could be missing out on revenue due to similar clinical errors and poorer outcomes.

By estimating the economic cost of burnout, and proposing solutions to the problem from an industry perspective, the veterinary profession can have a more tangible reason for addressing the issue from an organizational perspective (25). This work mirrors the approach to measure human physician burnout (9). This study explicitly estimates the cost of burnout and extrapolates the economic costs to the industry and individual veterinary clinic levels. Moreover, the heterogeneous costs of burnout are estimated for different types of practices (companion animal, food animal, mixed animal, and equine) to aid specific segments of the industry in addressing burnout.

## Materials and Methods

### Estimation Approach

The analysis looked at associate veterinarians in companion animal, food animal, mixed animal, and equine private practice. Similar in methods to the study conducted by Han et al. (9), this study focused on veterinary turnover and reduction in hours worked in a veterinary practice. Turnover and reduction in hours worked have a direct effect on the number of veterinarians needed to fill full-time equivalent positions to ensure that the demand in veterinary services is met. The output of the model is the cost associated with veterinarian burnout at the individual, and at the organizational level, by taking into account the difference between veterinarians who are burnt out and veterinarians who are not burnt out.

### Input Parameters and Data Sources

All costs in this analysis were adjusted for inflation to real 2020 United States dollars. The American Veterinary Medical Association (AVMA) Aptify membership database was used to obtain the number of veterinarians in the United States. This database is used in the calculations and simulations later in the methods to get industry level estimates. We use this database as most (~95%) US associate veterinarians are AVMA members and allows us to look at practice-type specific numbers. Numbers from the US Bureau of Labor statistics includes all non-clinical veterinarians and is not detailed enough to break about practice-type information. Several data sources were also used to compute calculations for the burnout calculator and described below.

#### Burnout Prevalence, Turnover, and Reduction in Work Hours

Data used to calculate burnout prevalence, turnover, and reduced work hours came from the 2016 to 2020 AVMA Census of Veterinarians surveys, administered annually in the late winter and spring by the Survey Research Laboratory of the University of Illinois at Chicago (2016–2019) and the Florida Agricultural Market Research Centre at the University of Florida, Food and Resource Economics Department. Response rates were 11.8% for 2016 (2,545 responses), 17.5% for 2017 (2,780 responses), 18.9% for 2018 (3,026 responses), 19.5% for 2019 (3,408 responses), and 22.0% for 2020 (3,556 responses). This survey was designed to gather information about the economics of veterinary practice and veterinary compensation asking veterinarians questions related to veterinary education, employment, compensation, debt, wellbeing, and demographics.

Burnout prevalence was measured using the Professional Quality of Life Scale (ProQOL) version 5 tool (26) and was included in the Census of Veterinarians surveys. It measures how one feels about his/her work as a professional caregiver. While the ProQOL tool was built for caregivers in a human health setting, it has also been used in veterinary medicine as well (19, 20, 27). The ProQOL tool measures compassion satisfaction and compassion fatigue (burnout and secondary traumatic stress) with 30 statements on how one feels about their work as a caregiver. Each respondent was asked to select on a 5-point Likert scale how they feel about each statement in the past 30 days, where 1 = never, 2 = rarely, 3 = sometimes, 4 = often, and 5 = very often (17). Ten statements were related to each of the following measures: compassion satisfaction, burnout, and secondary traumatic stress. For the purpose of this study only the burnout subscale questions are used to create burnout scores for each respondent. Ratings were summed (minimum score 10 and maximum score 50), and the summation of the scores were used in identifying the burnout threshold for this study. A minimum score of 25 was used to determine burnout prevalence among associate veterinarians (28).

Note that an alternative to the ProQOL scale is the Maslach Burnout Inventory (MBI) (29). The difference between the ProQOL and MBI is in the intended use of the scales. The MBI is only used to only measure burnout includes 22 questions that assess emotional exhaustion, depersonalization, and lack of personal achievement. Conversely, the ProQOL is a broader measure of professional satisfaction with burnout being one of three subscales. The data used for this study is a subset of a larger survey that was intended to answer a wide range of questions and the ProQOL scale and subscales aid in those overarching goals.

We directly observe turnover from survey respondents and also accounted for anyone who retired earlier than expected. Specifically, we observe those that have been working for their current employer for a year or less and if they had previously had a job. To estimate the intention to reduce clinical hours, veterinarians who desired to work fewer hours for less compensation were used. Reduced work hour intention was anyone who signaled that they desired to work fewer hours for less pay. Combining this information with each individual's rate of burnout allows for us to observe the conditional probability of reducing work hours (or turnover) based on burnout scores. Unfortunately, the actual reduction in hours is not observed through the survey, but this is also an issue in the physician study conducted by Han et al. (9). Thus, our measure acts as an upper bound on the estimate of cost for reducing work hours. Reduced work hour signaling is anyone who desired to work fewer hours and had a ProQOL burnout score <25. Reduced work hours signaling due to burnout is anyone who desired to work fewer hours and who had a ProQOL burnout score 25 or greater. Those that stated a reason for wanting to reduce work hours and fell into the burnout category often cited “burnout” or “working more hours than promised.” This suggests our conditional probability measure is relatively accurate, though still presents the analysis with a strong assumption.

#### Cost Parameters

Annual compensation numbers came from the 2016 to 2020 AVMA Census of Veterinarians where respondents indicated their compensation at the end of a given year. The replacement cost is derived from several research findings aggregated by the Center for American Progress in which employment positions that require higher education and specific training have higher replacement costs (30). The Society for Human Resource Management estimates replacement costs at 6–9 months of a former employee's salary (31, 32). Based on this information, a replacement cost of 66% was chosen for this study. Individual practice revenue numbers are provided by the 2017–2020 AVMA Practice Ownership Survey. This survey is distributed annually during the summer with response rates ranging from 8 to 16%. Average vacancy period is obtained from the AVMA Veterinary Career Center, looking at the 3-year average vacancy period from 2018 to 2020 which came to an average of 40 days, or a 5.7 weeks vacancy period. A summation of inputs and data sources are in Table 1.

**Table 1:** Study variable list and data sources.

**Input parameters**	**Data source**
Number of veterinarians (2020 estimates)	AVMA's Aptify membership database, year end 2020
**Prevalence/probabilities**	
Burnout proqol (≥25)	AVMA's census of veterinarians
Actual turnover (2020 data only)	AVMA's census of veterinarians
Actual turnover due to burnout (2020 data only)	AVMA's census of veterinarians
Reduce work hour (intention) P(RH = 1)	AVMA's census of veterinarians
Reduce work hour signaling P(RH = 1|B = 0)	AVMA's census of veterinarians
Reduce work hour signaling due to burnout P(RH = 1|B = 1)	AVMA's census of veterinarians
**Cost parameters**	
Median and mean annual salary for associates (2020 $ equivalents)	AVMA's census of veterinarians
Veterinarian replacement cost (66% of annual salary)—median and mean	Society for human resource management
Average net revenue contribution ($/associate vet/year)—median and mean	AVMA's practice ownership survey
Average vacancy period (days)	AVMA's veterinary career center

### Burnout Cost Calculations

Following the cost-consequence approach of Han et al. (9), the attributable cost of burnout is measured *via* turnover and reduced working hours. These two outcomes are chosen as they are organizational in nature, meaning they directly affect the businesses' profitability because they reduce clinical capacity. As previously mentioned, these two outcomes of burnout lead to reduced revenue generation for a veterinary clinic/hospital which is how the monetary cost is calculated. In this study, we simulate a hypothetical population of associate veterinarians (e.g., non-practice owners) that represent the current population of veterinarians participating in clinical practice. The particular focus of this study is on associates practicing in companion, equine, food, and mixed animal practices. As of 2020, the US population of associate veterinarians working in one of these four practice types was 68,532–55,952 in companion animal; 4,151 in equine; 4,291 in food animal; and 4,138 in mixed animal. The populations were used to simulate the overall costs of burnout to the veterinary service industry.

For the case of turnover, we directly observe associate veterinarian turnover as part of the 2020 questionnaire. Again, we observe those that have been working for their current employer for a year or less and if they had previously had a job. This eliminates recent graduates of veterinary medicine programs. We also observe those that retired from clinical practice early. Since we eliminate practice owners from the analysis this would not include those that sold a practice and chose to retire early. Since we directly observe turnover and burnout, we directly calculate the cost of burnout attributable to turnover for an individual as:


(1) $$Turnover\;Cost=c_{T}*P(T=1|B=1)$$

where *c*_*T*_ is the replacement cost associated with turnover (lost revenue/income, search costs, startup costs, training costs, etc.), and *P*(*T* = 1|*B* = 1) represents the conditional probability of turnover, *T*, given that the associate is experiencing burnout, as measured by the ProQOL tool. To simulate this for the industry, this cost is multiplied by the number of associate veterinarians overall or for the specific practice type. The cost of turnover is calculated from two specific components: replacement cost and lost income contribution due to position vacancy of an average of 40 days. Replacement cost is calculated as 66% of annual salary—we present both the mean and median from the data for transparency. Lost income contribution is the average net revenue contribution (average revenue contribution of a veterinarian to a practice minus the annual salary) multiplied by the average vacancy period as percent of the year.

The attributable cost of burnout due to reduced working hours is not directly captured in the survey data. Instead, we observe the intention to reduce working hours. As such, the calculation is expressed differently to account for the signaling of wanting to reduce work hours. Again, we follow the methodology set forth by Han et al. (9) and formulate the cost of reduced working hours for an individual as:


(2) $$\begin{array}{l} Reduced\;Hours\;Cost=c_{RH}\;*\;P(B\;=\;1)\\ \;*P(RH\;=\;1)\;*\;[P(RH\;=\;1|B\;=\;1)\\ \;-P(RH\;=\;1|B\;=\;0)] \end{array}$$

where *C*_*RH*_ is the cost of reduced working hours, *P*(*B* = 1) is the probability that the individual is experiencing burnout, *P*(*RH* = 1) is the probability that the individual is signaling a desire to reduce work hours, and *P*(*RH* = 1|*B* = 1)−*P*(*RH* = 1|*B* = 0) represents the difference in signaled intent for reduced working hours when burnout is present and absent, respectively (9). Again, to simulate this for the industry the cost is multiplied by the number of associate veterinarians overall or by specific practice type. The cost of reduced working hours is the percentage of the desired reduced working hours multiplied by the mean turnover cost.

#### Veterinary Technicians

Burnout within veterinary clinics goes beyond the veterinarian and affects all members of a clinical team. As such, since aggregate statistics are available, the economic cost of burnout is also estimated for veterinary technicians. In the case of technicians, only the rate of turnover due to burnout is known and not reduced working hours. While this is not ideal, the cost associated with turnover is much larger and captures a significant portion of the cost. The data on veterinary technicians comes from the National Association of Veterinary Technicians in America 2016 Demographic Results (33). Total number of veterinary technicians practicing in the industry is sourced from the Bureau of Labor Statistics (34). To account for average compensation, the Bureau of Labor Statistics median annual salary is used, while median revenue per non-veterinarian employee from the 2017 to 2020 AVMA Practice Ownership surveys is used to calculate the income loss from turnover. Average vacancy period for veterinary technicians was garnered from the AVMA's Veterinary Career Center. Veterinary technician turnover cost is calculated the same as that of veterinarians. The economic costs to the industry from burnout among veterinary technicians are added to the total cost of burnout from veterinarians to establish a total economic cost of burnout to the industry. While there are other staff members in clinical veterinary medicine that are also likely experiencing burnout, the data to calculate the economic cost for those staff members is currently unavailable.

### Sensitivity Analysis

For the purpose of brevity, the economic cost of burnout is calculated using measures of central tendency (mean/median) for many of the inputs. While this approach provides a singular answer, there is much variability given uncertainty around the inputs and the natural variability in the sample data. As such, a multivariate sensitivity analysis is performed to allow all inputs to vary and provide a range of possible outcomes. This presents a distribution of economic cost estimates and highlights the most probabilistic range of outcomes.

We used the @Risk version 8 program. This program allows inputs to vary along all possible values given a distributional assumption. The iterative process randomly pulls a value from the distributional range and simulates the outcome. This allows for a distribution of outcomes. Moreover, this approach allows for identification of the “most likely” range of outcomes so as to increase confidence in our estimates. However, it is important to note that the simulation is sensitive to the inputs and distributional assumptions. Since the probabilistic inputs on rates of burnout, turnover, and reduced working hours are calculated from data on veterinarians we can provide a robust, data informed approach to conducting the sensitivity analysis—we can use the data to inform the range and distribution of the inputs. For the inputs from secondary data sources, ranges are based on the reporting of the inputs and margins of error of 5%. Distributions are chosen to be either uniform or triangular distributions to represent relevant uncertainty about the input. All input ranges and distributional assumptions are reported in Table 2. Note that the sensitivity analysis is only performed for the data on veterinarians and not technicians. We do not perform sensitivity analysis on veterinary technicians as we do not have any data to inform the ranges of inputs.

**Table 2:** Multivariate sensitivity parameter input ranges.

**Inputs**	**Min**	**Med**	**Max**	**Distribution**	**Notes**
Burnout probability	1.04%	61.18%	72.89%	Beta	Range estimated based on different ProQOL Score cutoffs (max = 23, min = 42)
Actual turnover	10.00%	20.81%	30.00%	Triangular	Based on references (AAHA)
Actual turnover due to burnout	8.00%	13.06%	18.00%	Triangular	Based on references (AAHA)
Reduce work hour (Intention) P(RH = 1)	21.00%	25.96%	31.00%	Triangular	±5%
Reduce work hour signaling P(RH = 1|B = 0)	2.50%	7.46%	12.50%	Triangular	±5%
Reduce work hour signaling due to burnout P(RH = 1|B = 1)	13.80%	18.80%	23.80%	Triangular	±5%
Median annual salary (2020 $ equivalents)	$70,339.50	$88,902.00	$117,525.00	Pert	25th and 75th percentile
Mean annual salary (2020 $ equivalents)	$70,339.50	$104,869.00	$117,525.00	Pert	25th and 75th percentile
Replacement cost as percentage of annual income	0.5	66.00%	0.75	Uniform	Based on references
Average net revenue contribution ($/associate vet/year)—median	$110,410.50	$391,199.01	$520,975.00	Pert	25th and 75th percentile
Average net revenue contribution ($/associate vet/year)—mean	$110,410.50	$375,232.01	$520,975.00	Pert	25th and 75th percentile
Average vacancy period (days)	30	42	60	Uniform	Bounded between 1- and 2-month range
Average preferred reduction in clinical hours	0.25	48.12%	0.75	Triangular	25th and 75th percentile
Average preferred reduction in clinical hours associated with burnout	0.28	0.4986	0.75	Triangular	25th and 75th percentile

## Results

Using the median values and the entirety of the associate clinical profession, it is estimated that the economic cost of burnout due to turnover and reduced working hours is ~$997 million in lost revenue. The cost of turnover is almost twice as much as the cost of lost productivity from reduced working hours. In particular, the median cost of turnover per veterinarian is about $104,000 while the cost of reduced clinical hours at the average preferred reduction in working hours is about $56,000. Using mean values for turnover costs (e.g., salary and net revenue contribution), the industry level cost attributable to burnout is $1.075 billion as compared to $997 million.

**Figure 1:**
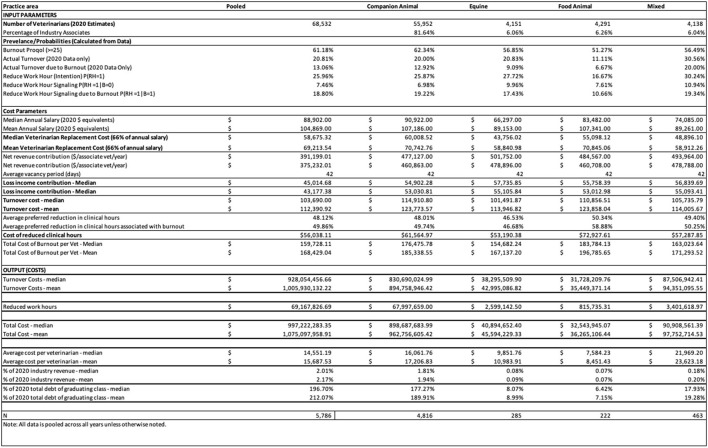
Burnout calculator for industry level analysis with all inputs and outputs.

**Figure 2:**
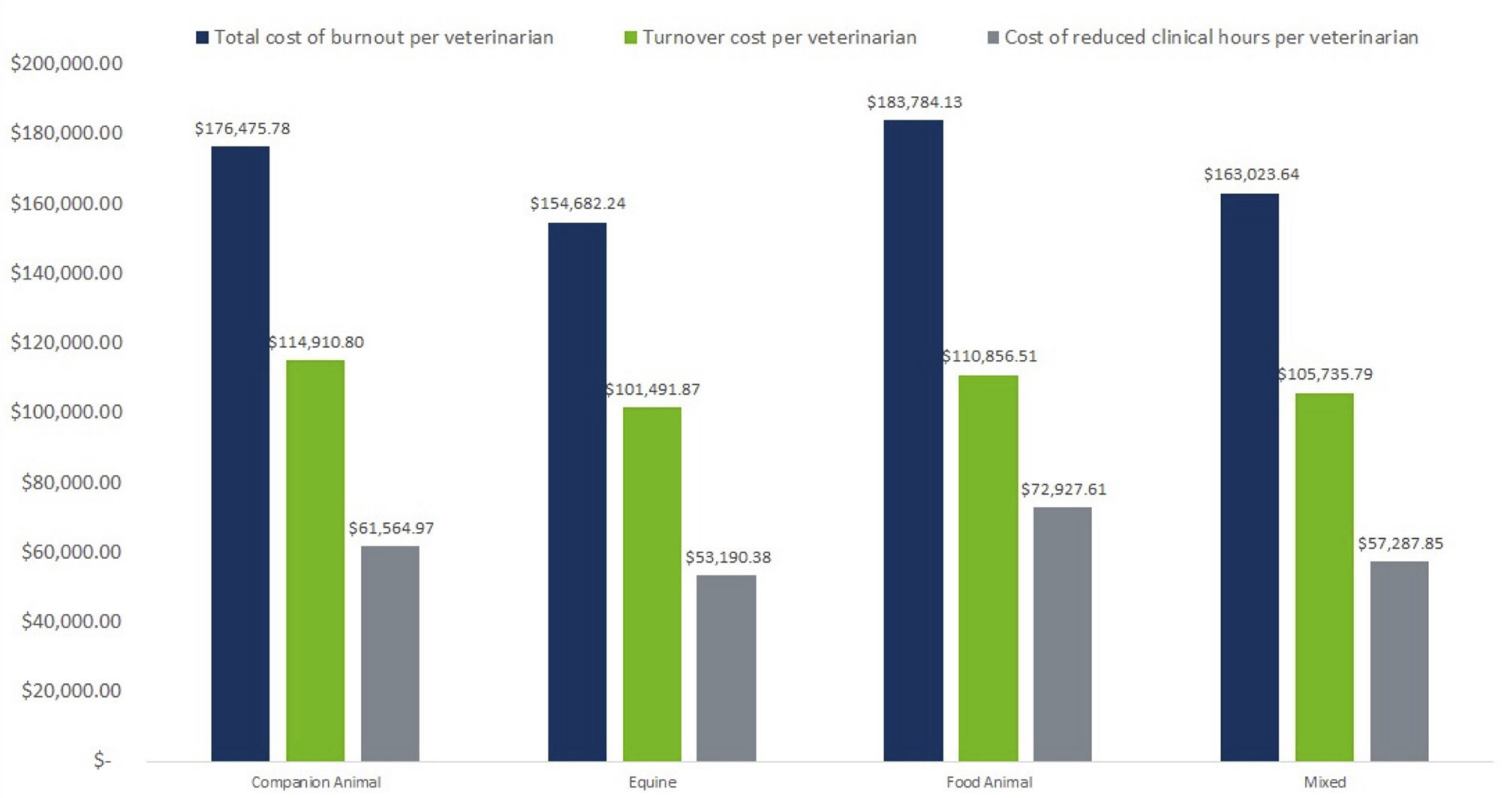
Attributable cost of burnout by practice type from turnover vs. reduced working hours.

To better understand the nuances of burnout cost across the industry, the same calculations are performed for each of four practice areas: companion animal, equine, food animal, and mixed animal. As seen in Figure 1, the practice type with the highest median cost of burnout per veterinarian are food animal veterinarians. Conversely, the practice type with the lowest burnout cost are equine veterinarians. However, as seen in Figure 2, companion animal veterinarians followed by mixed animal veterinarians contribute the most to total industry burnout cost. Food animal veterinarians have higher cost per veterinarian for both reduced working hours and turnover.

For veterinary technicians, the median industry cost of burnout attributable to turnover is about $933 million. The cost of turnover per veterinary technician, which is the only cost accounted for due to data limitations, is ~$59,000. The largest component of turnover cost for veterinary technicians is the loss of potential income given their absence, which is about $35,000. The replacement cost for veterinary technicians is estimated to be about $24,000. Adding the economic cost of burnout of veterinarians to that of veterinary technicians brings the total cost of burnout to a total of about $1.930 billion.

**Figure 3:**
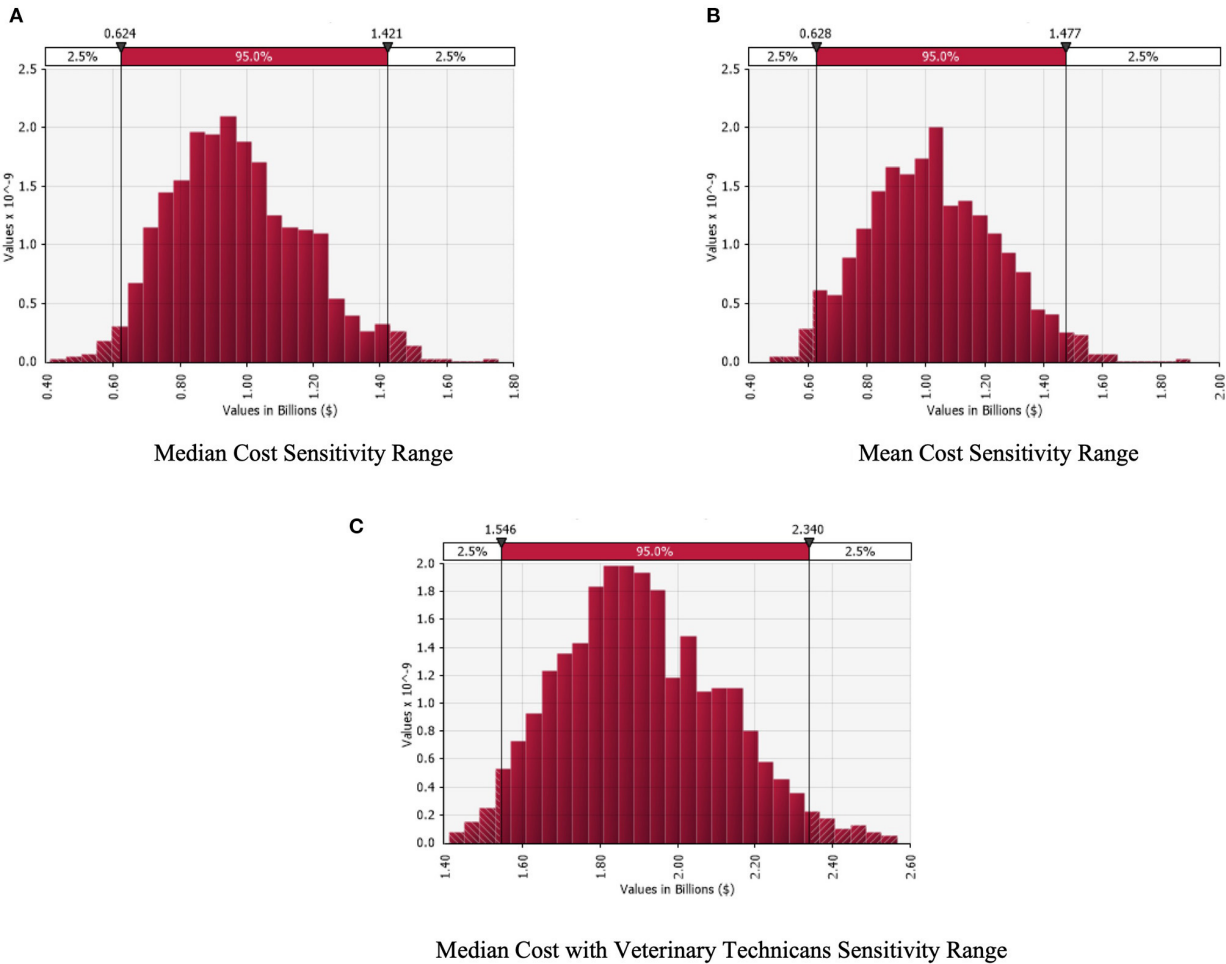
Multivariate sensitivity analysis for the economic cost of burnout under median **(A)**, Mean **(B)**, and addition of veterinary technicians **(C)** scenarios.

The multivariate sensitivity analysis, Figure 3, reveals the heavy amount of uncertainty within our estimates, but that the cost is still significant to the industry. The range of costs attributable to burnout using median values, Figure 3A, with 95% confidence intervals for veterinarians alone is $0.62 to $1.41 billion, while the mean range, Figure 3B, is $0.65 to $1.45 billion. When we add in the cost of veterinary technicians, the total attributable median cost of burnout, Figure 3C, to the industry ranges from $1.57 to 2.33 billion.

## Discussion and Limitations

Overall, this study adds to the growing literature on the effects of burnout within veterinary medicine and healthcare as a whole. The results suggests that the economic cost of burnout is large. Burnout among individuals within the veterinary industry has long been a concern, and this study provides tangible, economic reasons to induce actions aimed at reducing the prevalence of the problem.

As in Han et al. (9), these burnout estimates are likely a lower bound estimate since we only account for turnover and reduced working hours. There are other direct effects of burnout on the economic performance of firms as well as indirect effects such as the effect of turnover on patient care and other team members (35). Moreover, we do not consider the reduced productivity due to loss of clients or impacts to other aspects of veterinarian wellbeing such as compassion fatigue and secondary traumatic stress, among others. To place the aggregate cost of burnout into perspective, the total median cost is equivalent to about 2.01% of the veterinary service industry's value (36). Again, this is lost revenue and likely undervalued. This means that there are still several opportunities for the industry to expand in value. By helping to reduce burnout all members of a veterinary services team could see substantial benefits. By adding in the cost of veterinary technicians, the lost revenue is equivalent to 3.89% of the industry's value. From the sensitivity analysis, we find that the lower bound of the total industry cost just from veterinarians is at least 1% of total industry revenue.

While we conduct a multivariate sensitivity analysis, these estimates may not be reflective of individual clinics/hospitals. However, this calculator can be used to look at individual clinic/hospital costs given that the necessary information is being tracked. To aid in this process, we provide an editable version of the burnout calculator in the online Supplementary Material. The advantage of our study as compared to others in human medicine is that we are able to directly calculate the probability of turnover and reducing work hours conditional on burnout scores. When we compare burnout in veterinary medicine to other professions such as human healthcare providers, there is a clear, higher rate among veterinarians. Without intervention, turnover and desire to reduce working hours will continue to rise. This presents a threat to the long-term prosperity of the profession.

Our study has several limitations that should be noted. As previously mentioned, there are several other direct and indirect effects of burnout that have economic impacts on firms and the industry. While we provide a robust sensitivity analysis, our estimates are likely a lower bound estimate. This means the actual economic cost of burnout is much greater. Another limitation is our reliance on survey data to extract salary and revenue amounts. There is always a degree of sampling and measurement error in these estimates that are unavoidable. Measurement error may also exist in the measure of burnout for veterinary medicine used for this study (e.g., using ProQOL instead of MBI) (37). A third area of measurement area could arise from the use of a single measure of burnout, even if based on multiple questions/dimensions of the outcome. However, this reduction was necessary in order to reduce the complexity of the problem to a measure that can be used for economic analysis. Further, we do not have actual data on reduced working hours. For turnover, we estimate this percentage based on 2020 data only. This may be skewed due to the impact of the COVID-19 pandemic, though estimates are similar to those reported by the American Animal Hospital Association (38). For reduced working hours, we only measure the intention to reduce working hours and the associated amount. We provide a wide range for sensitivity estimates, but we do not observe the actual reduction in hours. While all of these limitations are of concern, we still have confidence that the true economic cost of burnout is much higher. As more data is collected, we plan to refine these calculations to prove true or not.

### Organizational Interventions to Burnout

It is imperative that the discussion of burnout include organizational interventions to address burnout. While there should always be a notion of individual self-care to combat burnout, clinics and hospitals also have a responsibility to reduce and prevent the causes of burnout. According to the AVMA (7), 40% of veterinarians are considering leaving the profession with the top reason for wanting to leave being a lack of work-life balance followed by mental health challenges such as stress, anxiety, and/or depression. From a recent wellbeing study (8), while wellbeing resources have been made available—many of which are online tools and resources—only 12% of veterinarians have accessed such resources. Although the creation and delivery of online tools and resources may be straightforward to provide, they have yet proven to have substantial impact on the wellbeing issues confronting the profession. This suggests a plethora of opportunities to implement organizational solutions to alleviate burnout. Moreover, organizational interventions can also have impacts on all aspects of veterinary team wellbeing and psychological health. As Pizzolon et al. (39) discuss, self-reported negative team environments increase incidence of burnout and other work-place stressors.

Human health physicians have been using organizational interventions to address burnout for several years. The interventions that have shown the most progress in reducing burnout and increasing work satisfaction focus on improving communication, optimizing workflow, and pursuing projects that target individual concerns (40). Some interventions include (1) scheduling monthly meetings with physicians focused on work-life/personal challenges or difficult patient care management issues; (2) improving workflow by offloading non-essential tasks to non-physician staff; (3) removing bottlenecks in patient rooms regarding medication reconciliation, vaccinations, and data entry; (4) reducing time pressure with plans for future increases in primary care visit time from 15 to 20 min; (5) instituting a new prescription line to free up nurse staff; and (6) presenting office work-life data as a platform to discuss issues within the clinic (40).

Shanafelt et al. (25) showed that lower leadership ratings—measured as employee reported ratings of current leadership across several dimensions—were associated with increased employee burnout, adding to the body of work showing that practice efficiency, expectations around workloads, and autonomy with an individual's work all impact wellbeing at the organizational level (41–45). Research evaluation by Panagioti et al. (15) revealed that interventions to address burnout were more successful at the organizational level than interventions at the individual clinician level. Specifically, strategies that focus on professional coping with stressors in the work environment were less successful than organizations minimizing or eliminating the stressors. Organizational interventions that use several types of mechanisms such as improved communication, optimizing workflows, and delegating special projects to team-members were most effective in reducing burnout. Changes in scheduling and reduction in an individual's workload were more commonplace in reducing burnout in the organization. Potential organizational solutions in the veterinary industry include, but are not limited to, rotational shifts like those in nurses and human medical physicians, limiting maximum work hours, and increasing the technician to veterinarian ratio. A focus on the workplace environment, staff and team culture in the practices, and quality of management and leadership are also important areas of focus from a wellbeing perspective.

Perhaps just as important as improving communication and workflow, another study found that good organizational leadership reduced burnout and stress among staff (25). In fact, better perceived leadership has an immediate and positive impact on reducing burnout. The dimensions of leadership that are important to improving burnout are skills that can be learned: (1) keeping people informed; (2) encouraging reports to suggest ideas for improvement; (3) providing feedback and coaching; (4) and recognizing quality job performance (25). Leadership is a key component to alleviating the burnout crisis in veterinary medicine and can have immediate benefits to individuals and the business. Moreover, quality leadership from the employee's perspective so that accurate assessments can be obtained.

Future work should focus on organizational interventions to the burnout problem. These solutions should be tailored to each individual practice, but the general ideas of quality leadership, improving workflow, feedback mechanisms, and increased utilization of current, non-veterinarian staff are all factors that can be implemented in the short-term. These short-term changes could have long-term impacts to reduce turnover and improve burnout for the entire profession. In essence, it is up to everyone (owners, veterinarians, technicians, and other staff) to decrease the barriers for a more satisfactory work experience and address this wellbeing crisis.

## Data Availability Statement

The data analyzed in this study is subject to the following licenses/restrictions: data is proprietary and owned by the American Veterinary Medical Association. Requests to access these datasets should be directed to Dr. Matthew Salois, msalois@avma.org.

## Author Contributions

CN and CH were responsible for data analysis and interpretation, drafting of the manuscript, and approval of the submitted manuscript. MS, CN, and CH were responsible for the conception of the study and manuscript writing and revisions. CH was responsible for acquisition of data and manuscript revision. All authors contributed to the article and approved the submitted version.

## Funding

This study was jointly funded by the American Veterinary Medical Association and Cornell University with support from Zoetis.

## Conflict of Interest

The authors declare that the research was conducted in the absence of any commercial or financial relationships that could be construed as a potential conflict of interest.

## Publisher's Note

All claims expressed in this article are solely those of the authors and do not necessarily represent those of their affiliated organizations, or those of the publisher, the editors and the reviewers. Any product that may be evaluated in this article, or claim that may be made by its manufacturer, is not guaranteed or endorsed by the publisher.

## Abbreviations

AVMA: American Veterinary Medical Association
ProQOL: Professional Quality of Life.
